# Help-seeking intention for mental illness and associated factors among Dessie town residents in Northeast Ethiopia

**DOI:** 10.1038/s41598-024-79656-w

**Published:** 2024-12-28

**Authors:** Habtam Gelaye, Atsedemariam Andualem, Abeba Beyene, Hailemariam Gezie

**Affiliations:** 1 Department of Psychiatry, College of Medicine and Health Sciences, Wollo University, 1145, Dessie, Ethiopia; 2 Department of Comprehensive Nursing, College of Medicine and Health Sciences, Injibara University, Injibara, Ethiopia; 3 Department of Emergency and Critical Care Nursing, College of Medicine and Health Sciences, Wollo University, Dessie, Ethiopia

**Keywords:** Help-seeking intention, Mental illness, Dessie Town residents, Psychology, Public health, Psychiatric disorders

## Abstract

Despite the increasing global burden of mental illness and the availability of effective evidence-based treatments, many individuals with mental illness do not seek professional help. Therefore, this study aimed to assess help-seeking intention for mental illness and associated factors among Dessie town residents, Northeast Ethiopia, 2021. A community-based cross-sectional study was conducted among 501 Dessie town residents. The data were collected by face-to-face interview. The General Help Seeking Questionnaire was used to assess help-seeking intention. Bivariable and multivariable logistic regression analysis techniques were used. Finally, a statistically significant level was declared at a p-value less than 0.05. Among 501 participants, 67.5% were likely to seek help from health professionals. Being female (AOR = 4.695, 95% CI = 1.63– 13.50), being single (AOR = 0.330, 95% CI = 0.12–0.89), and having good knowledge (AOR = 3.030, 95% CI = 1.25–7.35) were significantly associated with help-seeking intention. This study indicated that the participants’ help-seeking intention was inadequate. Sex, marital status, monthly income, and knowledge of mental illness were found to be associated with help-seeking intentions for mental illnesses. Therefore, community healthcare workers, healthcare administrators, and religious and community leaders should work to enhance the help-seeking intention of the community.

## Introduction

Mental health problems are increasingly prevalent, affecting approximately 450 million people worldwide, and the impact of these problems is significant on an individual and the national level^1,2^. According to the World Health Organization’s (WHO) Global Burden of Disease Collaborator study, mental health disorders are known to represent two of the ten prominent causes of disability and account for 35.6% of the total burden of disease^3^. Mental illnesses affect the social, economic, academic, occupational, and recreational aspects of the functionalities of victims, family members, caregivers, and the whole community.

Professional help-seeking intention refers to an individual’s perceived likelihood of seeking assistance from health professionals^4^. Mental health help-seeking intentions are an adaptive coping mechanism where individuals attempt to obtain external help if they believe they are mentally ill. This response typically involves consulting a doctor, mental health professional, psychologist, or social worker^5–7^. It can be influenced by factors such as personal beliefs, social norms, perceived illness severity, and the accessibility of health services^8,9^ The mental health literacy of the community can also directly affect the help-seeking intention for mental illness from health professionals. People with good mental health literacy have better help-seeking intentions and vice versa^10–12^.

Despite a variety of management options available, around two-thirds of people with a known mental disorder worldwide do not seek help from health professionals^13–15^. A research review conducted in developing countries indicates that supernatural causes of mental disorders are more widely believed and traditional sources of help, such as spiritual healers, are preferred over medical advice for a variety of mental health problems^16^.

A study conducted in Rwanda revealed that only 36.0% of participants received help from a healthcare unit^17^. Another study done in Butajira, Ethiopia, also shows only 41% of the society preferred seeking help from health institutions^18^. A study conducted in Jimma town also showed that the most frequently visited source of help was the informal help sources^19^. Moreover, a study conducted at the holy water site of Gebremenfes Kidus Church, Ethiopia, showed that most of the respondents had help-seeking behavior from traditional forms of help like religious leaders, holy water sprinkling, and individuals who believed to have a special power of knowing mental illness and prescribing traditional treatment for mental illness^8^.

In the study area, many healthcare institutions are providing mental health services. However, evidence-based data are scarce regarding the status of the help-seeking intention of the community for mental illnesses and mental health service utilization in Dessie town and Northeast Ethiopia at large. Therefore, this study aimed to assess the help-seeking intention and identify the factors associated with mental health help seeking among the residents of Dessie town in North East Ethiopia.

## Methods and materials

### Study area and period

The study was conducted in Dessie town, Northeast Ethiopia. The town has 5 sub-cities and 26 (18 urban and 8 rural) kebeles with a total population of 296, 966. There are two governmental and four private hospitals, seven governmental health centers, and other private clinics in the town. The study was conducted from 1st December 2020 to 30th January 2021.

### Study design, population, and eligibility criteria

A community-based cross-sectional study was conducted among residents of Dessie town. All individuals available during the data collection period were considered the study population. Those who were willing to participate in the study and who had lived in the town for the last six months were included in the study, whereas individuals who were unable to respond due to illness at the time of data collection and those individuals with an age below 18 years were excluded.

### Sample size and sampling procedure

#### Sample size

A single population proportion formula was used to calculate the sample size, taking the proportion of 81.5%^6^ to obtain the maximum sample size at 95% CI and 5% marginal error. The sample was calculated as follows:

n = (Z α/2)^2^ p (1-p) / d^2^.

n = (1.96)^2^ (0.815) (1-0.815)/ (0.05)^2^ = **232**.

Where, n = required sample size; Z α/2 = z value at α which is 1.96; p = prevalence of help-seeking intention (81.5%); d = margin of error (5%).

After we added a 10% non-response rate, the sample size became **255**. Because we used a multi-stage sampling technique, we multiplied the sample size by design effect (2) to reduce the role of confounders and errors.

255*2 = 510; thus, the final sample size was **510**.

#### Sampling procedure

**Fig. 1:**
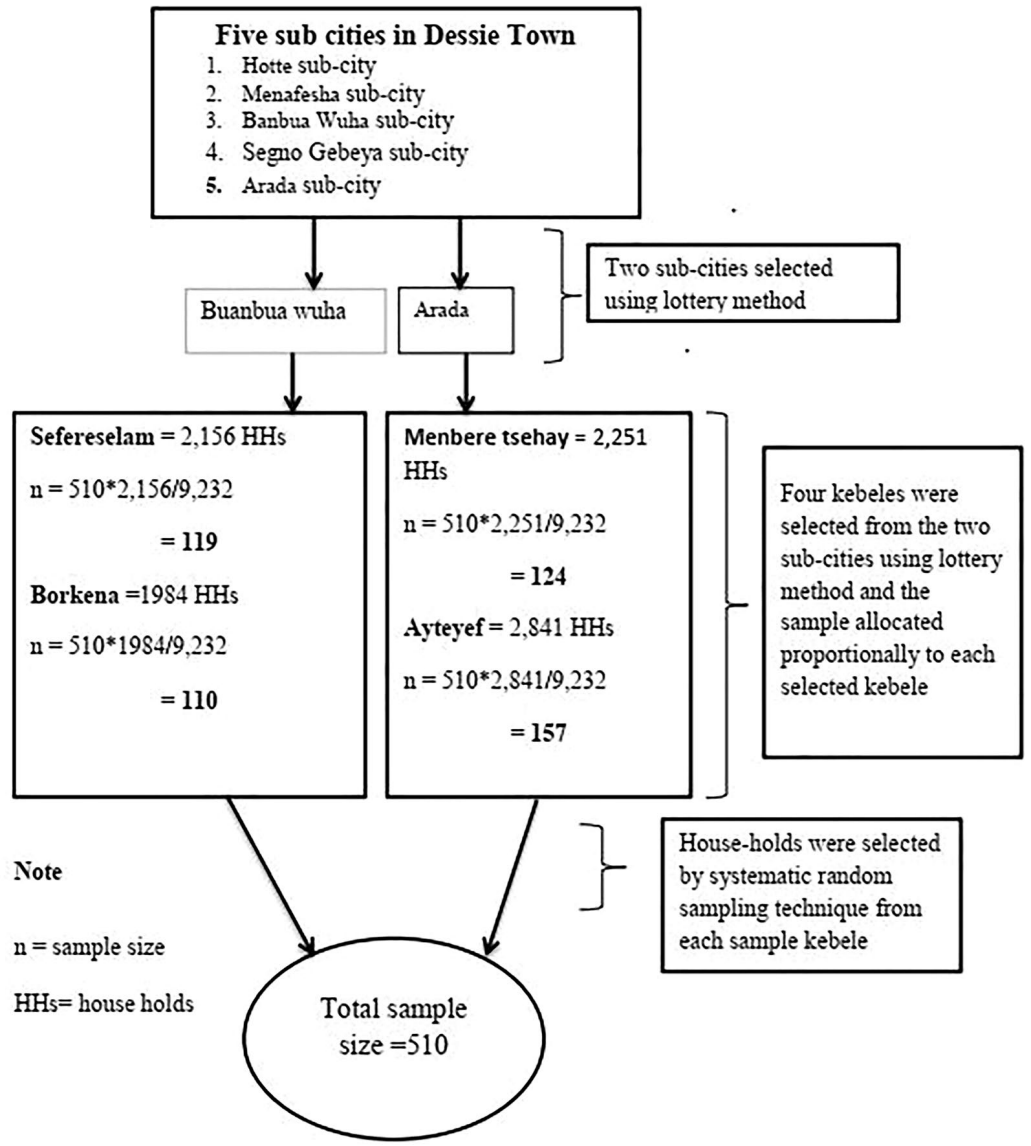
Schematic representation of sampling technique showing the number of samples from each selected kebele of Dessie town, 2021.

The study participants were selected using a multi-stage sampling technique. In the first stage, two sub-cities (Buanbua Wuha and Arada) were selected by the lottery method from the five sub-cities in the town. In the second stage, four kebeles (two from each selected sub-city) were also selected by the lottery method. Thirdly, based on their household size, a proportional allocation of the sample was done to each selected kebele. Finally, the households were selected using a systematic random sampling technique in every 18th household (Fig. 1). The first household was selected randomly and continued every 18th interval until the required sample was obtained. One of the adults in each household was included in this study. If there were more than one adult in the household, the data collectors selected one adult by lottery method.

### Study variables

#### Dependent variables

Mental health help-seeking intention.

#### Independent variables

Socio-demographic characteristics, attitude, perceived experience, knowledge of the mental illness, behavior, perceived severity of mental illness, illness perception about mental illness, social support, self-stigma, and substance-related factors.

### Operational definitions

#### Mental health help-seeking intention

Participants intended to seek help from health workers for personal or family mental illness when they thought they had a problem^6^.

#### Attitude towards mental illness

Respondents who scored ≤ the mean score of the Attitude Towards Seeking Professional Psychological Help Scale (ATSPPHS) were considered to have an unfavorable attitude, and those who scored above the mean were considered to have a favorable attitude towards mental illness^20^.

#### Knowledge of mental illness

Respondents who scored above the mean score of the Mental Health Knowledge Schedule (MAKS) were grouped as having good knowledge, and those who scored ≤ the mean were considered to have poor knowledge^21^.

#### Reported behavior

participants who scored above the mean score of the first four questions of the Reported and Intended Behavior Scale (RIBS).

#### Intended behavior

Participants who scored above the mean score of the next four questions of the Reported and Intended Behavior Scale (RIBS).

#### Self-stigma

Participants who scored above the mean score of the Self-Stigma of Seeking Help Scale (SSOSHS).

#### Poor social support

Those respondents scored 3–8 and were considered to have poor support using the Oslo-3 Social Support Scale (OSS-3)^22^.

### Data collection tools and procedures

#### Data collection tools

The data were collected using a structured questionnaire adapted from previous studies^6,20–22^. The questionnaire had nine parts. The first part was about the socio-economic and demographic characteristics of participants. The second part was about participants’ previous experiences and perceptions of mental illness. The third part was the Oslo social support scale (Oslo-3), which measures the respondents’ social support level. The fourth part was about the substance use habits of respondents. The fifth part was the Mental Health Knowledge Schedule (MAKS) tool to assess participants’ mental health knowledge. The sixth part was the Attitude Towards Seeking Professional Psychological Help Scale (ATSPPHS) tool to assess participants’ attitudes toward professional help-seeking. The seventh was the Reported and Intended Behavior Scale (RIBS), the eighth part was about the Self-Stigma of Seeking Help Scale (SSOSHS), and the ninth part was the General Help Seeking Questionnaire to assess participants’ level of help-seeking intention. All scales were validated in Ethiopia, and for our use, we translated from English to Amharic by a language expert and back to English by another expert during analysis.

### Data collection procedure

The data were collected by four psychiatry nurse professionals with a Bachelor of Science degree qualification and supervised by one mental health specialist with a Master of Science degree qualification. A one-day training was given to the data collectors and the supervisor about the objectives of the study, the nature of each variable, the way of approaching the study participants, and other issues. The data were collected by face-to-face interviews after obtaining written informed consent from each participant.

### Data quality control

One-day training was given to data collectors and supervisors. A pre-test was done among 10% of the sample size two weeks before the beginning of the actual data collection. Based on the pretest findings, necessary revisions were made to the questionnaire. Moreover, during the data collection, data collectors were strictly supervised. At the end of each data collection day, the questionnaires were checked for completeness and appropriateness. To ensure the quality of our study, we consulted senior mental health specialists and checked the internal consistency of the data collection tool by computing Cronbach’s alpha from pretest findings. The Cronbach’s alpha value of the tool was 0.83.

### Data processing and analysis

The data were checked, cleaned, coded, entered into Epi-data 3.1, and exported to Statistical Package for Social Sciences (SPSS) version 26 for analysis. Descriptive statistics were presented in frequency and percentage using tables and figures. Bivariable logistic regression was done for all independent variables. Only variables with a p-value < 0.25 were eligible for multivariable logistic regression analysis because our objective was not to highlight the theoretical or clinical significance of the independent variables^23,24^. After multivariable logistic regression, variables with a p-value < 0.05 were considered statistically significant.

## Results

### Socio-economic and demographic characteristics of respondents

From a total sample size of 510, 501 participants completed the interview, which gave a response rate of 98.62%. About 38.3% of participants were in the age group of 25–34 years, and 53.5% were male. Most of the study participants (97.4%) were ethnically Amhara, and 61.5% were Orthodox Christians. Half of the respondents (49.5%) were employed, and more than half of the participants (58.9%) had a monthly income of ≥ 1201 ETB (Table 1).

**Table 1:** Socio-economic and demographic characteristics of the study participants among Dessie town residents (*n* = 501) Dessie, Ethiopia, 2021.

Variables	Variables category	Frequency(*n*)	Percent (%)
Age	18–24	98	19.6
	25–34	192	38.3
	35–44	100	20.0
	≥ 45	111	22.2
Sex	Male	268	53.5
	Female	233	46.5
Marital status	Married	207	41.3
	Single	199	39.7
	Widowed	46	9.2
	Divorced	49	9.8
Ethnicity	Amhara	488	97.4
	Oromo	2	0.4
	Tigrie	7	1.4
	Others^a^	4	0.8
Religion	Orthodox	308	61.5
	Muslim	172	34.3
	Protestant	21	4.2
Educational status	Uneducated	29	5.8
	Read and write	18	3.6
	Primary	96	19.2
	Secondary	172	34.3
	Higher education	186	37.1
Occupational status	Employed	248	49.5
	Unemployed	253	50.5
Income	≤ 750	112	22.4
	751–1200	94	18.8
	≥ 1201	295	58.9

Others = Gurage, Agew and Afar.

### Previous experience and perception of mental illness

Most of the participants (94.6%) had never suffered from mental illness, and about 64.1% of them knew someone who had a mental illness. 33.7% of the participants witnessed someone hurt by individuals with mental illness. 41.3% of the participants categorized mental illness as very severe. 66.3% of the participants responded that psychosocial factors cause mental illness. 94.2% of participants believe that mental illness requires treatment (Table 2).

**Table 2:** Previous experience and perception about mental illness among Dessie town residents (*n* = 501) Dessie, Ethiopia, 2021.

Variables	Variables category	Frequency	Percent (%)
Have you ever suffered from mental illness?	No	474	94.6
	Yes	27	5.4
Do you know someone who has mental illness?	No	180	35.9
	Yes	321	64.1
If “yes” for the above question, what is your relation?	Relative	59	11.8
	Neighbor	84	16.8
	Friend	54	10.8
	Other^a^	124	24.8
Have you been involved in caring people who have mental illness?	No	175	34.9
	Yes	146	29.1
Have you ever been hurt by people who have mental illness?	No	264	52.7
	Yes	57	11.4
Do you witness someone hurt by people who have mental illness?	No	152	30.3
	Yes	169	33.7
How severe do you think mental illness is?	Mild	15	3.0
	Moderate	38	7.6
	Severe	241	48.1
	Very sever	207	41.3
What is the cause of mental illness?	Psychosocial	332	66.3
	Physical	56	11.2
	Spiritual	13	2.6
	Genetic	42	8.4
	Others^b^	58	11.6
Do you think the mental illness requires treatment?	No	29	5.8
	Yes	472	94.2

Others^a^ = street people, colleagues…etc; Others^b^ = evil spirit, evil eye.

### Social-support, substance-related, knowledge, attitude, behavior, and self-stigma-related characteristics

Only 20.6% of the participants had strong social support. Regarding substance use, 31.7% of the participants ever used substances in their life. Among these, 16.6% and 10.2% used khat and alcohol, respectively. 24.8% were also current substance users. About 39.7% of the participants had poor knowledge of mental illness. Only 58.3% of the participants had a favorable attitude toward seeking professional help for mental illness. 48.5% of the participants had self-stigma (Table 3).

**Table 3:** Social support, Substance-related, knowledge, attitude, behavior, and self-stigma-related characteristics of participants among Dessie town residents (*n* = 501) Dessie, Ethiopia, 2021.

Variables	Variables category	Frequency	Percent (%)
Level of social Support	Poor	180	35.9
	Moderate	218	43.5
	Strong	103	20.6
Have you ever used any substance?	No	342	68.3
	Yes	159	31.7
If “yes” which type of substance?	Tobacco	25	5.0
	Alcohol	51	10.2
	Khat	83	16.6
Have you ever used any substances in the past 3 months?	No	35	7.0
	Yes	124	24.8
If “yes” which type of substance?	Tobacco	25	5.0
	Alcohol	49	9.8
	Khat	50	10.0
Knowledge	Poor knowledge	199	39.7
	Good knowledge	302	60.3
Attitude	Unfavorable	209	41.7
	Favorable	292	58.3
Past or reported behavior	No reported behavior	316	63.1
	Reported behavior	185	36.9
Intended behavior	No intended behavior	218	43.5
	Intended behavior	283	56.5
Self- stigma	No self- stigma	258	51.5
	Self-stigma	243	48.5

### Help-seeking intentions from health professionals and other sources for mental illness

**Fig. 2:**
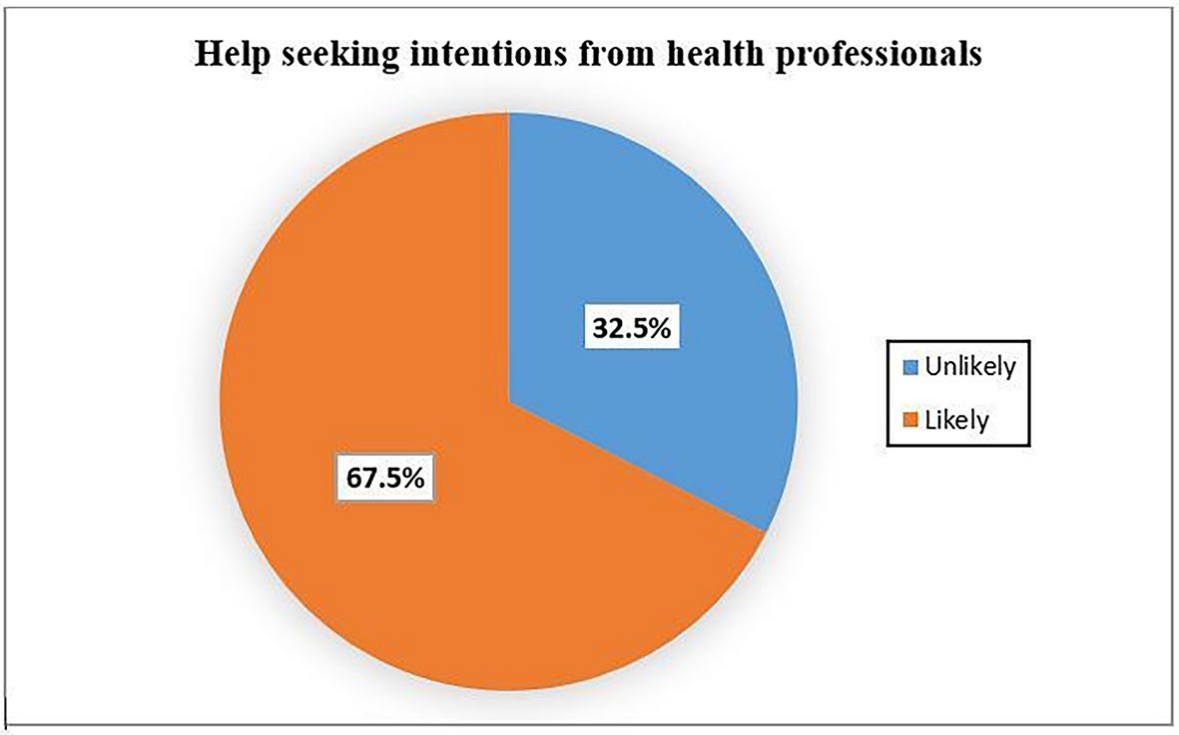
Intention to seek help from health professionals among Dessie town residents (*n* = 501), Ethiopia, 2021.

**Fig. 3:**
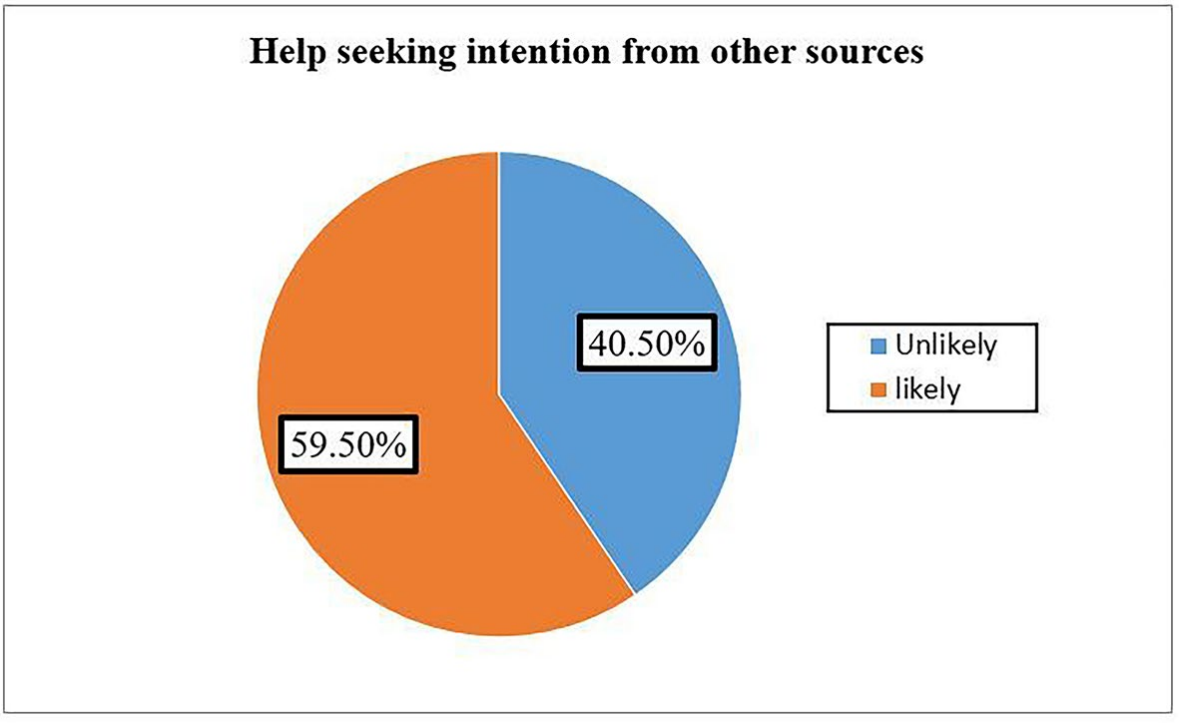
Intention to seek help from other sources of help among Dessie town residents (*n* = 501), Ethiopia, 2021.

Among the total respondents, 67.5% (95% CI of 63.3-71.7%) were likely to seek help from health professionals. 59.5% were also likely to seek help from other sources (Fig. 2) and (Fig. 3), respectively.

### Factors associated with the help-seeking intention for mental illness from health professionals

All variables with a p-value < 0.25 in the bivariable logistic regression analysis were candidates for multivariable logistic regression analysis. On multivariable logistic regression, sex, marital status, income, and knowledge were found to be statistically significant factors of help-seeking intention for mental illness. We did not include all variables with a p-value ≥ 0.25 because the objective of the study was not to highlight the theoretical or clinical significance of variables^23,24^.

Female respondents were 4.7 times more likely to have mental health help-seeking intention for mental illness (AOR = 4.695, 95% CI = 1.63–13.49) compared to males. Participants with a monthly income of ≥ 1201 ETB were 3 times more likely to have mental health help-seeking intention for mental illness (AOR = 3.233, CI = 1.15–9.08) compared to those who have a monthly income of < 750 ETB. Additionally, participants who have good knowledge were 3 times more likely to have mental health help-seeking intention for mental illness (AOR = 3.030, CI = 1.25–7.35) compared to those who have poor knowledge. On the other hand, being single was also a statistically significant factor of help-seeking intention for mental illness, in which there were 67% less likely to seek help from health professionals (AOR = 0.330, 95% CI = 0.12–0.89) than married participants (Table 4).

**Table 4:** Factors associated with help-seeking intention for mental illness from health professionals among Dessie town residents, Ethiopia, 2021.

Variables		COR(95% CI)	AOR(95% CI)	*P* -value
Sex	Male	1	1	
	Female	1.733(1.18–2.54)	4.695(1.63–13.49)	0.004*
Marital status	Married	1	1	
	Single	0.786(0.52–1.20)	0.330(0.12–0.89)	0.028*
	Widowed	0.933(0.47–1.87)	--	--
	Divorced	0.544(0.29–1.03)	--	--
Monthly income	≤ 750 ETB	1	1	**--**
	751–1200 ETB	1.282(0.75–2.21)	--	--
	≥ 1201 ETB	2.217(1.42–3.50)	3.233(1.15–9.08)	0.026*
Have you suffered from mental illness?	No	1	--	**--**
	Yes	0.584(0.27–1.28)		
Do you think the mental illness requires treatment?	No	1	--	**--**
	Yes	2.721(1.28–5.80)	--	**--**
Level of social support	Poor	1	--	**--**
	Moderate	1.308(0.86–1.98)	--	**--**
	Strong	1.588(0.94–2.70)	--	**--**
Knowledge	Poor	1	1	**--**
	Good	2.141(1.46–3.14)	3.030(1.25–7.35)	0.014*
Attitude	Unfavorable	1	**--**	**--**
	Favorable	1.881(1.29–2.75)	**--**	**--**
Intended behavior	No	1	**--**	**--**
	Yes	0.744(0.51–1.09)	**--**	**--**

* = *p* < 0.05.

## Discussion

This study aimed to assess help seeking intention for mental illness and associated factors among residents of Dessie town, and it found 67.5% of the residents had mental health help seeking intention. It also identified different factors significantly associated with the help-seeking intention.

The study found that 67.5% (95% CI of 63.3-71.7%) of the respondents had the intention to seek help for mental illness from health professionals. This finding was supported by two community-based cross-sectional studies conducted in Aykel town^25^ and Gondar Zuria District^26^ of Ethiopia, which found that 71.2% and 63.8% of participants had an intent to seek help from any health professionals. This similarity might be because of using a similar assessment tool (GHSQ), similarity in study design, and similarities in study participants’ socioeconomic and demographic as well as religious characteristics. The healthcare system is also similar among the study areas.

This finding was higher than two community-based studies done in Jimma town^19^ and Seka Chekorsa district of Jimma zone^27^ Ethiopia, which found that only 49.4% and 38.8% of the participants sought help for common mental health problems, respectively. This difference might be due to differences in sample size, data collection tool (the Jimma town study used the Actual Help Seeking Questionnaire), and participants’ differences, in which 33.6% of Jimma town study participants were mentally ill and the Seka Chekorsa study participants were rural residents. There is also cultural variation between the participants of the above studies and the current study.

This finding was also higher than studies done in Rwanda^10^, Greece^20^, Switzerland^21^, and the USA^22^, which revealed 36.0%, 50–60%, 22.5%, and 33.4% of the respondents sought help from healthcare professionals and healthcare units, respectively. This discrepancy might be due to the differences in the general population of the four countries and differences in sample size. The Rwanda study was done among two population groups. The Greek study used the Inventory of Attitude towards Seeking Mental Health Services (IASMHS) tool, and the majority of the participants were females. The other reason might also be the differences in study design, in which the Swiss study was a retrospective follow-up study involving mainly adults in the age group of 16–40 years and mentally ill patients.

On the other hand, this study’s finding was lower than studies conducted in Mertule Mariam town, Ethiopia^6^, in which 81.5% of respondents had a help-seeking intention from healthcare workers; a cross-sectional survey study conducted in China^28^ among a rural adult population that revealed approximately 80% of the participants were willing to seek psychological help if needed; and a study conducted in Japan^29^, which reported 85.9% of participants would seek help from formal sources. The possible reasons for this difference might be socio-demographic and cultural variations among participants, sample size, data collection tool, and methodological differences. The health literacy of the Chinese and Japanese might be higher than Ethiopians. The healthcare system might also be better in China and Japan compared to Ethiopia.

Variables that had a significant association with help-seeking intention for mental illness from health professionals were female sex, being single, monthly income of ≥ 1201 ETB, and good knowledge of mental illness.

Female respondents were 4.7 times more likely to have mental health help seeking intention for mental illness compared to males. This finding was in line with studies conducted in Ethiopia^19^, Australia^30^, China^28^, Japan^29^, and the USA^31^, which demonstrated that being female was a positive predictor of help-seeking intention. This consistency might be because of similarities in study design, analysis method, and assessment tool (GHSQ). The other possible reasons might be more positive attitudes concerning psychological openness in women compared to men, as well as gender-role differences where men’s traditionally advantaged social status and greater power may make it more difficult to seek help for mental health issues^32^. Moreover, culturally, men’s masculinity is demonstrated through power, dominance, self-control, and self-reliance, in which expressions of psychological distress, and related help-seeking behaviors might be regarded as a lack of masculinity.

Participants who were single in marital status were 67% less likely to seek help for mental illness from health professionals compared to single once. This finding was supported by the studies conducted in Ethiopia^19^ and China^33^, in which married participants had significantly more positive help-seeking intentions for mental disorders than single ones. This consistency might be due to similarity in study design. However, this finding was inconsistent with two Ethiopian studies conducted in Aykel town^25^ and Mertule-Mariam^6^ and the study conducted in the USA^31^, which revealed participants who were not in a relationship had greater help-seeking intention. The possible reasons for this inconsistency might be sample size variation (both studies used a larger sample size than the current study), and the Aykel town study was aimed to assess help-seeking intention for depression only. The other reason for the decreased intention to seek help for mental illness from health professionals among single participants might be that a single person could not get any parental, familial, or spouse role of advising, pushing, helping, or supporting to seek help for mental illness from health professionals.

Participants who had a monthly income of ≥ 1201 ETB were 3.23 times more likely to seek help for mental illness compared to participants who had a monthly income of ≤ 750 ETB. This study finding is supported by the study conducted in Japan^29^, which demonstrated lower income was associated with decreased likelihood of help-seeking intentions. This is because people who have enough money might have access to different media and other information and can be able to get help from health professionals. A person whose income is good can also afford healthcare costs.

Furthermore, respondents who had good knowledge were 3.03 times more likely to seek help from healthcare professionals compared to those who had poor knowledge. This finding was supported by studies conducted in China^28^, England^34^, and four European region populations^35^ that higher or better mental health knowledge was a positive predictor of help-seeking intention. This might be because a person with a higher level of knowledge can recognize the signs and symptoms of mental illness early and can understand the benefits of mental health treatments and options. A person with good mental health knowledge can also avoid the embarrassment of seeking help for mental illness from healthcare professionals.

### Strengths and limitations of the study

This was the first study in Northeast Ethiopia, particularly in Dessie town, which reported the residents’ intent to seek mental health help and associated factors. The study had an adequate sample size, which was representative of the population and supports the generalizability of the findings. However, the study had limitations. One of the possible limitations of this study could be its cross-sectional nature, in which it does not show a cause-and-effect relationship. Social desirability biases may also exist, leading respondents to report more socially desirable responses that may not accurately reflect real-life experiences. The other limitations might also be related to the interview techniques, and variables with a p-value ≥ 0.25 were not included in the analytical approach. There might also be recall bias for some variables from participants.

### Implications and feature direction

Despite the availability of many healthcare facilities that are providing mental health services, the mental health help-seeking intention of Dessie town residents is still inadequate. This implies different strategies have to be designed and implemented to enhance the mental health help-seeking intention of the community.

Regarding clinical and community practices, implementing gender-based interventions, mental health education programs, and utilizing different peer support models are important to enhancing the community help-seeking intention^36,37^.

Policymakers should also develop mental health awareness campaigns targeting urban and rural populations, integrate mental health services with primary healthcare facilities, and support mental health research projects with adequate funding^36–40^.

Because this research has limitations, future research, such as longitudinal studies, which can explore cause-and-effect relationships, and qualitative studies, which can investigate barriers to mental health help-seeking intention, like cultural influences from the community, healthcare workers, and health managers’ perspectives, is required.

### Conclusion and recommendations

The findings of our study indicate that only two-thirds (67.5%) of the participants had an intent to seek help for mental illnesses, which indicates the mental health help-seeking intent of Dessie town residents was inadequate. This result is even lower than the findings of some of the previous studies done in Ethiopia. Additionally, being female, having a monthly income of ≥ 1200 ETB, and having good knowledge were found to be positively associated with the participants’ mental health help-seeking intention. On the other hand, being single in marital status was found to be negatively associated with help-seeking intentions for mental illnesses.

Therefore, health practitioners, community healthcare workers, healthcare administrators, religious and community leaders, and other stakeholders should consider implementing strategies to increase mental health knowledge and mental health literacy through face-to-face trainings for the community and primary health workers, national and local level community and school based campaigns, use of technologies such as online delivery of trainings and consultations, and dissemination of information via printed and audio-visual materials^36,37,39,41^ to enhance the mental health help-seeking intention of the community. Integrating mental health literacy in the educational system has also a positive effect on the help-seeking intention for mental health problems^42^. There should also be activities to improve economic burden of the community help seeking intention for mental illness. The government and other stakeholders shall subsidize mental health services and expand health insurance coverages, improving employment opportunities and income security, and support community based mental health initiatives^38,43^.

## Data Availability

All the data regarding the findings are presented in the manuscript, and any additional raw data are accessible from the corresponding author for reasonable request.

## Abbreviations

AOR: Adjusted Odd Ratios
ATSPPHS: Attitude towards Seeking Professional Psychological Help Scale
CI: Confidence Interval
CMD: Common Mental Disorder
COR: Crude Odd Ratios
GBD: Global Burden of Disease
GHSQ: General Help-Seeking Questionnaire
ICCMH: Integrated Clinical and Community Mental Health
LAMICs: Low and Middle-Income Countries
MAKS: Mental Health Knowledge Schedule
RIBS: Reported and Intended Behavior Scale
SPSS: Statistical Package for Social Science Studies
SSOSHS: Self-Stigma of Seeking Help Scale
WHO: World Health Organization

## References

[1] Alemayehu, N. Bipolar disorder in rural Ethiopia Community-based studies in Butajira for screening, epidemiology, follow-up, and the burden of care. (2009).

[2] Saraceno, B. Invited papers the WHO World Health Report 2001 on mental health. *Epidemiol. e Psichiatr Soc.* **11**, 83–89 (2017). 10.1017/s1121189x00005546 12212469

[3] Metrics, G.H. Global, regional, and national incidence, prevalence, and years lived with disability for 354 diseases and injuries for 195 countries and territories, 1990–2017 : a systematic analysis for the global burden of Disease Study 2017. *Glob Heal Metrics*. **392**, 1990–2017 (2018). 10.1016/S0140-6736(18)32279-7 PMC6227754 30496104

[4] Lally, J., Mcdonald, C. & July Stigma of mental illness and help-seeking intention in university students. (2014). 10.1192/pb.bp.112.041483

[5] Wilson, C. J., Rickwood, D. & Deane, F. P. Depressive symptoms and help seeking in young people. *Clin. Psychol.* **11**(3), 98–107 (2007).

[6] Yeshanew, B., Belete, A. & Necho, M. Help - seeking intention and associated factors towards mental illness among residents of Mertule Mariam town, East Gojam Zone, Amhara Region, Ethiopia: a mixed - method study. *Ann. Gen. Psychiatry* **19**, 1–11 (2020). 32127907 10.1186/s12991-020-00261-y PMC7045738

[7] Li, X. Y. et al. Predictors of professional help-seeking intention toward depression among community-dwelling populations: A structural equation modeling analysis. *Front. Psychiatry* **13**, 1–12 (2022). 10.3389/fpsyt.2022.801231 PMC8907597 35280177

[8] Hailemariam, K. W. Perceived causes of Mental Illness and Treatment seeking behaviors among people with Mental Health problems in Gebremenfes Kidus Holy Water Site. *Am. J. Appl. Psychol.* **3**, 34–42 (2015).

[9] Clement, S. et al. What is the impact of mental health-related stigma on help-seeking? A systematic review of quantitative and qualitative studies. *Psychol. Med.* **45**, 11–27 (2015). 24569086 10.1017/S0033291714000129

[10] Kim, E. J., Yu, J. H. & Kim, E. Y. Pathways linking mental health literacy to professional help-seeking intentions in Korean college students. *J. Psychiatr Ment Health Nurs.* **27**, 393–405 (2020). 31954091 10.1111/jpm.12593

[11] Jung, H., von Sternberg, K. & Davis, K. The impact of mental health literacy, stigma, and social support on attitudes toward mental health help-seeking. *Int. J. Ment Health Promot*. **19**, 252–267 (2017).

[12] Mohammadi, A. Q., Johnston, L. & Ojha, K. Mental health literacy among Afghan adults: A community-based cross-sectional survey study in Herat city. *Razi Int. Med. J.* **3**(1), 36–43 (2023).

[13] Levav, I. & Rutz, W. The WHO World Health Report 2001: New understanding - new hope. *World Heal Organ.* **3**, 19–44 (2002). 12013710

[14] Thornicroft, G. Most people with mental illness are not treated. *Lancet*. **370**, 807–808 (2007). 17826153 10.1016/S0140-6736(07)61392-0

[15] Henderson, C., Evans-Lacko, S. & Thornicroft, G. Mental illness stigma, help seeking, and public health programs. *Am. J. Public. Health*. **103**, 777–780 (2013). 23488489 10.2105/AJPH.2012.301056 PMC3698814

[16] Tibebe, A. & Tesfay, K. Journal of depression and anxiety public knowledge and beliefs about mental disorders in developing Countries: A review. *J. Depress Anxiety* **S3-004**, 1–4 (2015).

[17] Umubyeyi, A., Mogren, I., Ntaganira, J. & Krantz, G. Help-seeking behaviours, barriers to care and self-efficacy for seeking mental health care : a population-based study in Rwanda. *Soc. Psychiatry Psychiatr Epidemiol.* **51**, 81–92 (2016). 26433379 10.1007/s00127-015-1130-2 PMC4720720

[18] Alem, A., Jacobsson, L., Araya, M., Kebede, D. & Kullgren, G. How are mental disorders seen and where is help sought in a rural Ethiopian community ? *Acta Psychiatr Scand.* **100**, 40–47 (1999). 10470354 10.1111/j.1600-0447.1999.tb10693.x

[19] Kerebih, H., Abera, M. & Soboka, M. Pattern of help seeking behavior for Common Mental disorders among urban residents in Southwest Ethiopia. *Qual. Prim. Care*. **25**, 208–216 (2017).

[20] Taylor, S. M. & Dear, M. J. Scaling Community attitudes toward the mentally III. *Schizophr Bull.* **7**, 226–240 (2015). 10.1093/schbul/7.2.225 7280561

[21] Rüsch, N. Knowledge and attitudes as predictors of intentions to seek help for and disclose a Mental illness. *Psychiatr Serv.* **62**, 675 (2011). 21632739 10.1176/ps.62.6.pss6206_0675

[22] Abiola, T., Udofia, O. & Zakari, M. Psychometric properties of the 3-Item Oslo Social Support scale among clinical students of Bayero University Kano, Nigeria. *Malaysian J. Psychiatry*. **22**, 32–41 (2013).

[23] Zhang, Z. Model building strategy for logistic regression: purposeful selection. 4:4–10. (2009). 10.21037/atm.2016.02.15 PMC4828741 27127764

[24] Ziaul, M., Chowdhury, I. & Turin, T. C. Variable selection strategies and its importance in clinical prediction modelling. (2020). 10.1136/fmch-2019-000262 10.1136/fmch-2019-000262 PMC7032893 32148735

[25] Shumet, S. et al. Intention to seek help for depression and associated factors among residents of Aykel town, Northwest Ethiopia: cross - sectional study. *Int. J. Ment. Health Syst.* **13**, 1–8 (2019). 30962818 10.1186/s13033-019-0274-y PMC6434882

[26] Getaneh, E., Kassie, A., Shetie, B., Wolde, M. & Yigzaw, N. Perceived cause and determinants of help-seeking behavior of schizophrenia among Gondar Zuria district residents, Northwest Ethiopia. *Heliyon*. **7**, e07212 (2021). 34159271 10.1016/j.heliyon.2021.e07212 PMC8203707

[27] Tesfaye, Y., Agenagnew, L., Tucho, G. T. & Anand, S. Attitude and help-seeking behavior of the community towards mental health problems. 1–13. (2020). 10.1371/journal.pone.0242160 PMC7660493 33180818

[28] Yu, Y. et al. Mental Health help-seeking intentions and preferences of rural Chinese adults. *PLoS One*. **10**, 1–16 (2015). 10.1371/journal.pone.0141889 PMC4636424 26545095

[29] Suka, M., Yamauchi, T. & Sugimori, H. Relationship between individual characteristics, neighbourhood contexts and help-seeking intentions for mental illness. *BMJ Open.* **5**, 1–9 (2015). 10.1136/bmjopen-2015-008261 PMC4538253 26264273

[30] Calear, A. L., Batterham, P. J. & Christensen, H. Predictors of help-seeking for suicidal ideation in the community: risks and opportunities for public suicide prevention campaigns. *Psychiatry Res.* **219**, 525–530 (2014). 25048756 10.1016/j.psychres.2014.06.027

[31] Parent, M. C., Hammer, J. H., Bradstreet, T. C., Schwartz, E. N. & Jobe, T. Men’s Mental Health help-seeking behaviors: an intersectional analysis. *Am. J. Mens Health*. **12**, 64–73 (2018). 29226771 10.1177/1557988315625776 PMC5734540

[32] Judd, F., Komiti, A. & Jackson H. How does being female assist help-seeking for mental health problems? *Aust. N. Z. J. Psychiatry* **42**(1), 24–29 (2008). 18058440 10.1080/00048670701732681

[33] Chin, W. Y., Chan, K. T. Y., Lam, C. L. K., Lam, T. P. & Wan, E. Y. F. Help-seeking intentions and subsequent 12-month mental health service use in Chinese primary care patients with depressive symptoms. 1–10. (2015). 10.1136/bmjopen-2014-006730 PMC4316433 25631313

[34] Psychia-, G. & Crespigny, D. Knowledge and attitudes as predictors of intentions to seek help for and disclose a Mental illness. *Psychiatr Serv.* **62**, 675–878 (2011). 21632739 10.1176/ps.62.6.pss6206_0675

[35] Kohls, E. et al. Public attitudes toward depression and help-seeking: impact of the OSPI-. *J. Affect. Disord*. **217**, 252–259 (2017). 28437762 10.1016/j.jad.2017.04.006

[36] Broek, M. et al. Interventions to increase help-seeking for mental health care in low- and middle-income countries: A systematic review. 1–29. (2023). 10.1371/journal.pgph.0002302 PMC10499262 37703225

[37] Brown, J. S. L. et al. *How Can We Actually Change Help-Seeking Behaviour for Mental Health Problems among the General Public ?* (Development of the ‘ PLACES ’ Model, 2022). 10.3390/ijerph19052831 PMC8909998 35270523

[38] Castillo, E. G. et al. Community Interventions to Promote Mental Health and Social Equity. (2019). 10.1007/s11920-019-1017-0 PMC6440941 30927093

[39] Kelly, C. M., Jorm, A. F. & Wright, A. Improving mental health literacy as a strategy to facilitate early intervention for mental disorders Claire. 187. (2007). 10.5694/j.1326-5377.2007.tb01332.x 17908021

[40] Xu, Z. et al. Effectiveness of interventions to promote help-seeking for mental health problems: systematic review and meta-analysis. (2018). 10.1017/S0033291718001265 29852885

[41] Johnson, J. A. & Sanghvi, P. Technology-Based Interventions to Improve Help-Seeking for Mental Health Concerns: A Systematic Review. 44. (2022). 10.1177/02537176211034578 PMC9301737 35949632

[42] Kutcher, S. et al. A school mental health literacy curriculum resource training approach : effects on Tanzanian teachers’ mental health knowledge, stigma and help - seeking efficacy. *Int. J. Ment. Health Syst.* **10**, 1–9 (2016). 27493684 10.1186/s13033-016-0082-6 PMC4973111

[43] Knapp, M. et al. Economic barriers to better mental health practice and policy. (2006). 10.1093/heapol/czl003 10.1093/heapol/czl003 16522714

